# Global up-to date emissions using the EDGAR Fast-Track methodology

**DOI:** 10.1038/s41597-025-04806-2

**Published:** 2025-07-16

**Authors:** Diego Guizzardi, William Becker, Federico Pagani, Marilena Muntean, Monica Crippa

**Affiliations:** 1 European Commission, Joint Research Centre (JRC), Ispra, Italy; 2 Unisystems S.A., Milan, Italy

**Keywords:** Climate-change mitigation, Environmental impact

## Abstract

One of the goals of the Paris Agreement is to regularly collect knowledge on the collective effort of abating global greenhouse gas (GHG) emissions. However, national emission inventories submitted to the UNFCCC only partly provide this information, due to the limited time series completeness and time lag compared to the current year. Developing up-to date global GHG emission inventories is therefore essential to enable the assessment of global GHG emission trends and to contribute to the 5-years cycle of the global carbon stocktake as foreseen by the Paris Agreement. The Emissions Database for Global Atmospheric Research (EDGAR) fills this knowledge gap, providing on an annual basis reliable and up-to date independent estimates of global CO_2_ and non-CO_2_ GHG emissions with a minimum delay (t-1), for all countries and sectors. This work describes the approach developed and applied within EDGAR to compute emissions up to the most recent year, thus making EDGAR a reliable and timeliness source of information for assessing the collective effort towards climate targets.

## Background & Summary

In light of the limited remaining carbon budget and the relevance of recent global emission trends^1–3^, up to date information on global, regional and country specific greenhouse gas (GHG) emissions is essential. GHG emission estimates for all anthropogenic sectors are needed for achieving global climate commitments and for effective policy-making. The national emission inventories (NEIs) submitted by the Parties under the United Nations Framework Convention on Climate Change (UNFCCC) represent the ‘official’ GHG emission reporting and are typically available on an annual basis with two years delay (up to t-2) for Annex I countries^4^. However, for some non-Annex I countries, policy makers and scientists have to rely on national communications and biennial update reports which can have a delay of several years in emission reporting^5^, as shown in Fig. S1. Under the Enhanced Transparency Framework of the Paris Agreement, Parties are required to submit biennial transparency reports (BTR) every two years, with the first submission due by 31 December 2024. The reporting of National Emission Inventories using Common Reporting Tables may facilitate the compilation of global GHG emissions up to t-2 in the upcoming years. An overview of current available inventories, their methodologies, imitations, and how they can be used in support of policy making is provided by NASEM^6^ and Perugini *et al*.^7^.

One of the goals of the Paris Agreement is to regularly gather knowledge on the collective effort of abating global GHG emissions. This is done in 5-year cycles, with the compilation of the global carbon stocktake (CGST) starting in 2023^8^. The enhanced transparency framework (ETF) of the Paris Agreement^9^ also fosters the development of best practices, knowledge and sharing experience on GHG emission reporting to enable the assessment of the global collective effort towards climate targets. In this context, the global emission inventory work developed since the 1990s by the Emissions Database for Global Atmospheric Research^10^ represents a reference when developing global emission inventories following the Intergovernmental Panel on Climate change (IPCC) methodology for GHG inventory compilation^11–13^. Since 2012, EDGAR has demonstrated its reliability and timeliness in providing global CO_2_ and non-CO_2_ GHG emissions on an annual basis with a minimum delay (t-1)^14–16^. The EDGAR GHG emissions estimates represent an independent source of information, which complements official reporting under the UNFCCC, thus supporting the ETF and completing the global picture needed to assess the collective effort towards climate targets under the GCST. It also offers knowledge and experience for countries with limited statistical infrastructure and expertise in compiling GHG emission inventories. Consequently, EDGAR global GHG emissions estimates are used as the reference dataset for historic emissions in the Sixth Assessment report of the IPCC^1^, but also contribute annually to the UNEP Emission Gap Reports^3,17–19^ and support the international climate negotiations of the European Commission^20,21^. Moreover, the EDGAR data are instrumental for climate policy research, as shown for example by Stechemesser *et al*.^22^.

EDGAR is a global bottom-up emission inventory of greenhouse gas and air pollutants, which covers historic time series from 1970 until t-1, all countries and all anthropogenic emitting sectors, with the exception of Land Use, Land Use Change and Forestry (LULUCF). EDGAR emissions are computed following a consistent IPCC methodology based on activity data and emission factors (mostly with a Tier 1 or Tier 2 approach) for all countries and sectors, thus allowing comparability among these estimates. In order to compute GHG emissions, international statistics are used as input information for the activity data, thus representing the essential element to be regularly updated to most recent years to provide up to date emissions. EDGAR methodology and data sources are fully described in Janssens-Maenhout *et al*.^23^ and Crippa *et al*. However, two main activity data sources used by EDGAR are the energy balances of the International Energy Agency^24^ and agricultural statistics from the Food and Agriculture Organisation^25^, which overall cover more than 85% of global GHG emissions. This is why we dedicate specific focus in this work on how extending these statistics up to the year t-1. The Fast-Track assumptions for the remaining sectors are described in section 2.4 and Table 4. Some of these sectors do not require an extrapolation since yearly updates based on stable statistics are performed, while the remaining ones are extrapolated using 5-year trends.

This article describes a globally applicable methodology to compute GHG emissions up to the most recent years (t-1), which is applied to the EDGAR global emission inventory, but can be extended to any other national/regional/global emission dataset. Starting from an emission inventory developed accordingly with the IPCC methodology (e.g. EDGAR) or an inventory whose emission sources can be allocated to IPCC related sectors, we provide here the concept and data sources to extend the baseline emissions up to t-1. This work addresses one of the main challenges when compiling global emission inventories, which is associated with the lack of readily available detailed international statistics to compute accurate, timely and up to date (t-1) GHG emissions. These limitations are present also in several other global GHG inventories, such as those produced by the International Energy Agency^26^, Energy Institute^27^, the Global Carbon Budget project^28^ or by the Community Emissions Data System^29^. These emission inventories mainly focus on the fossil CO_2_ component, provide emissions up to t-2 and partly rely on the EDGAR estimates as well. Furthermore, we provide an assessment of the accuracy of this extrapolation methodology comparing projected emissions with those obtained using robust statistics.

## Methods

Emissions of GHGs and air pollutants are estimated in EDGAR accordingly with the following formula:

$${{EM}}_{i\left(C,t,x\right)}=\sum _{j,k}{{AD}}_{i(C,t)}\ast {{TECH}}_{i,j\left(C,t\right)}\ast {{EOP}}_{i,j,k\left(C,t\right)}\ast {{EF}}_{i,j(C,t,x)}\ast {(1-{RED})}_{i,j,k(C,t,x)}$$

with emissions (EM) from a given sector i in a country C accumulated during a year t for a chemical compound x, with the country-specific activity data (AD) quantifying the human activity for sector i, the mix of j technologies (TECH, varying between 0 and 1), the mix of k (end-of-pipe) abatement measures (EOP, varying between 0 and 1) installed with a share k for each technology j, and the uncontrolled emission factor (EF) for each sector i and technology j with relative reduction (RED) by abatement measure k. Emissions are typically computed for around 165 IPCC detailed sectors and around 60 fuel types. Activity data information are typically retrieved from international statistics, such as the International Energy Agency Energy Balances, the Food and Agriculture Organisation livestock and crop statistics, data from the USGS geological surveys, etc. A comprehensive description of the EDGAR emission computation methodology and data sources can be found in Janssens-Maenhout *et al*.^23^, Crippa *et al*.^30^, and Oreggioni *et al*.^31,32^. An IPCC-based methodology has been also applied to compute GHG emission uncertainty by sector for the EDGAR estimates, as detailed in Solazzo *et al*.^33^.

In order to extend emission time series up to the year t-1 in the absence of robust statistical data, a Fast-Track (FT) procedure is developed and implemented within the EDGAR database. This procedure aims to extend the activity data underlying the corresponding emissions to the latest years, making use of aggregated statistical information (e.g. by fuel type, by region, etc.) which is available beyond the latest year (typically t-3) of complete international statistics. The approach of using a Fast-Track methodology to extend fossil CO_2_ emissions to the latest years was already introduced by Myhre *et al*.^34^. However, in this work we aim at covering all GHG and air pollutant emissions from all sectors, providing details on how to incorporate data from the latest international statistics.

The general approach of the FT methodology is to leverage activity data that are available up to t-1, but at a higher level of aggregation (e.g. macro fuel categories, individual vs. regional data, no sector specifications, etc.) than the normal level of reporting in EDGAR. For example, national (or regional) coal consumption statistics are available up to t-1, but the detailed disaggregation into activity sectors and coal type (e.g. bituminous coal, sub-bituminous coal, lignite, anthracite, etc.) is only available up to t-3. The FT approach involves calculating the mapping of the disaggregated statistics relative to the aggregated statistics at t-3, then using the same mapping to disaggregate the aggregated statistics at t-1 and t-2. The implicit assumption here is that the mapping remains constant. This assumption is not limiting the correct quantification of total CO_2_ and GHG emissions from fossil fuel combustion since it only depends on the carbon content of the fuel and the combustion process producing CO_2_. However, it may have an impact on the relative sector contribution to total emissions. When no aggregated data sources are available to perform this mapping, the time series of certain sectors are used as proxies for other sectors, or a 5-year trend may be calculated.

In this section, we provide the data sources and methodology for each anthropogenic emitting sector to extend GHG emission time series up to t-1. This procedure is applied to the EDGAR database to the activity data and not to the emissions estimates (which are calculated based on activity data), thus offering the opportunity to consistently compute CO_2_ and non-CO_2_ GHG emissions as well as short-lived climate forcers (SLCFs). However, in particular when dealing with SLCFs, we recommend caution due to the relevance of the penetration of new technologies and abatement measures which may strongly influence emissions in the most recent years.

### Combustion sources

The EDGAR database relies on IEA energy balance statistics as activity data for fossil fuel combustion sources^15^. These fuel consumption statistics are extended for the years t-2 and t-1 using the annual change rate calculated from the Energy Institute detailed statistics by fuel type and country or region^27^, while still assuming the same sectoral breakdown as in the last year of the IEA energy balance statistics (see Table 1). Consequently, the emissions calculated for the Fast-Track years (t-2 and t-1) may differ from subsequent updated data by the IEA and require revisions on a yearly basis. In order to improve the accuracy of t-2 emissions, the inclusion of IEA Energy Balances released in April every year may be considered, as described in Crippa *et al*.^35^. This April pre-release provides consolidated statistics up to the year t-2 for combustion related sectors for 62 countries (OECD countries, Argentina, Brazil, Chile, China, India, South Africa, etc.) covering around 60% of fossil CO_2_ emissions from combustion activities. This update also allows reducing differences between EDGAR GHG emission estimates of two consecutive years. For biofuel combustion, the trend for most recent years is obtained at country level using the latest FAOSTAT data (for solid biofuels)^25^ and from EI^27^ for liquid biofuels across all sectors. Industry Statistics (fuel consumption) from IATA^36^ are used to extend international aviation data. Table 1 summarises all the sources and metrics used to extend the activity data of combustion related sources.

**Table 1:** Drivers and data sources to extend the time series of combustion related sectors.

Sector	IPCC 2006 category	Metric used to extend the time series	Data source
Power generation (fossil fuels)	1A1a	Electricity generation from oil, coal and gas	EI, 2023
Power generation (liquid biofuels)	1A1a	Consumption of biodiesel, biogasoline and bioliquids	EI, 2023
Power generation (solid biofuels)	1A1a	Consumption of primary solid biomass and charcoal	FAOSTAT, 2023
Combustion in manufacturing industry (fossil fuels)	1A2	Consumption of coal, natural gas, oil	EI, 2023
Combustion in manufacturing industry (liquid biofuels)	1A2	Consumption of biodiesel, biogasoline and bioliquids	EI, 2023
Combustion in manufacturing industry (solid biofuels)	1A2	Consumption of primary solid biomass and charcoal	FAOSTAT, 2023
Road and off-road transport (fossil fuels)	1A3b, 1A3c, 1A3e	Consumption of coal, natural gas, oil	EI, 2023
Road and off-road transport (liquid biofuels)	1A3b, 1A3c, 1A3e	Consumption of biodiesel, biogasoline and bioliquids	EI, 2023
Road and off-road transport (solid biofuels)	1A3b, 1A3c, 1A3e	Consumption of primary solid biomass and charcoal	FAOSTAT, 2023
Small scale combustion (fossil fuels)	1A4, 1A5	Consumption of coal, natural gas, oil	EI, 2023
Small scale combustion (liquid biofuels)	1A4, 1A5	Consumption of biodiesel, biogasoline and bioliquids	EI, 2023
Small scale combustion (solid biofuels)	1A4, 1A5	Consumption of primary solid biomass and charcoal	FAOSTAT, 2023
Domestic aviation	1A3a	Consumption of Jet/Kerosene	EI, 2023
International Aviation	1A3a	Fuel consumption	IATA, 2023
Domestic shipping	1A3d	Consumption of Fuel Oil	EI, 2023
International Shipping	1A3d	Consumption of Fuel Oil	EI, 2023

### Fuel exploitation sources

GHG emissions associated with the extraction, processing and transformation of solid, liquid and gaseous fuels are typically derived from IEA energy balance statistics^24^. In order to extend these data up to most recent statistics, Energy Industry^27^ production data by fuel type and country are used. Table 2 summarises the main drivers used to compute the trends for the years 2021 and 2022 for each sub-sector related with fuel exploitation and distribution. Venting and flaring data do not require extrapolation being entirely available up to t-1^15^.

**Table 2:** Drivers and data sources to extend the time series of fuel exploitation and distribution sectors.

Sector	IPCC 2006 category	Metric used to extend the time series	Data source
Production of coal	1B1a	Production of coal	EI, 2023
Production of crude oil	1B2a, 1B2c	Production of crude oil and condensate	EI, 2023
Production of natural gas liquids	1B2a, 1B2c	Production of natural gas liquids	EI, 2023
Production of natural gas	1B2b	Production of natural gas	EI, 2023
Production of biofuels (liquid)	1B2a, 1B2c	Production of biofuels	EI, 2023
Refinery and transformation of fossil fuels (combustion)	1A1b, 1A1c	Consumption of coal, natural gas, oil and liquid biofuels	EI, 2023
Refinery and transformation of solid biofuels (combustion)	1A1b, 1A1c	Consumption of primary solid biomass and charcoal	FAOSTAT, 2023
Transformation of fuels (fugitive)	1B1b, 2C1b	5 years trend, trend based on iron and steel production	—
Oil transmission and distribution	1B2a, 1B2c	5 years trend	—
Gas transmission and distribution	1B2b	5 years trend	—

### Agricultural sources

For agricultural sources, World Agricultural Production data from the United States Department of Agriculture USDA^37^ are used to extend FAOSTAT statistics up to t-1. Statistical information on aggregated crops type (yield and/or area for Barley, Mixed Grain, Corn, Rice, Rye, Sorghum, Wheat) and livestock category (diary and non-diary cattle, pigs) by country or main world producers are considered to compute trends, as summarised in Table 3. Since USDA reports data only for the main contributors to global emissions, the number of countries for which the USDA statistics are available and the corresponding GHG emissions fraction is also reported in Table 3.

**Table 3:** Drivers and data sources to extend the time series of agriculture related sources.

Sector	IPCC 2006 category	Metric used to extend the time series	Data source	Number of countries	Fraction of global emissions
Agricultural soils and burning of crop residues: Barley cultivation	3C4, 3C1	Crop yield of barely	USDA, 2023	90	99.96%
Agricultural soils and burning of crop residues: Corn cultivation	3C4, 3C1	Crop yield of corn	USDA, 2023	140	99.84%
Agricultural soils and burning of crop residues: Rice cultivation	3C7, 3C1	Crop yield and area of rice	USDA, 2023	Yield: 97 Area: 96	94.99% 95.79%
Agricultural soils and burning of crop residues: Wheat cultivation	3C4, 3C1	Crop yield of wheat	USDA, 2023	115	99.99%
Agricultural soils and burning of crop residues: Oats cultivation	3C4, 3C1	Crop yield of oats	USDA, 2023	59	99.53%
Agricultural soils and burning of crop residues: Rye cultivation	3C4, 3C1	Crop yield of rye	USDA, 2023	48	97.14%
Agricultural soils and burning of crop residues: Sorghum cultivation	3C4, 3C1	Crop yield of sorghum	USDA, 2023	77	98.39%
Agricultural soils and burning of crop residues: all other cultivation	3C4, 3C1	Crop yield of mixed grain	USDA, 2023	24	4.75%
Enteric fermentation, pasture, manure management and animal waste as fertiliser of dairy cattle	3A1, 3C4, 3A2, 3C4	Dairy cattle stock number	USDA, 2023	71	42.50%
Enteric fermentation, pasture, manure management and animal waste as fertiliser of non-dairy cattle	3A1, 3C4, 3A2, 3C4	Non-dairy cattle stock number	USDA, 2023	71	50.54%
Enteric fermentation, pasture, manure management and animal waste as fertiliser of pigs	3A1, 3C4, 3A2, 3C4	Pigs stock number	USDA, 2023	57	65.72%
Enteric fermentation, pasture, manure management and animal waste as fertiliser of all other livestock categories	3A1, 3C4, 3A2, 3C4	5 years trend	—	—	—
Use of nitrogen fertilisers for agricultural soils	3C4	5 years trend	—	—	—
Agricultural soils: liming application	3C2	Trend based on lime production	—	—	—
Agricultural soils: urea application	3C3	5 years trend	—	—	—
Indirect N_2_O emissions from agricultural sources and atmospheric deposition of NOx and NH3	3C5, 3C6, 5A	No extrapolation	—	—	—

In this work, we do not provide information on how to extrapolate data for the Land Use, Land Use Change and Forestry sector. We recommend the use of Global Wildfire Information System data^38^ for wildfires including their annual updates, while for the other LULUCF components, the extrapolation method may require different assumptions depending on the methodology used (e.g. carbon stock changes, gain and losses, complex land use modelling, etc.).

### Industrial processes and other sources

Around 4% of global GHG emissions can be estimated on an annual basis up to most recent years (t-1) making use of robust statistics. Therefore, no Fast-Track procedure is applied to these components. In particular, this is the case of CO_2_ emissions from industrial processes such as Cement production, Lime production, Iron and Steel and sinter production and Ferroalloys production (see Table 4 and Crippa *et al*.^15^).

**Table 4:** Methodology for calculating industrial processes and other source emissions up to t-1.

Sector	IPCC 2006 category	Metric used to extend the time series
Flaring	1B2C	No extrapolation
Cement production	2A1	No extrapolation
Lime production	2A2	No extrapolation
Iron and Steel and sinter production	2C1	No extrapolation
Ferroalloys production	2C2	No extrapolation
Indirect N_2_O emissions from the atmospheric deposition of nitrogen in NOx and NH_3_	5A	No extrapolation
Fossil fuel fires	5B	No extrapolation
Glass Production	2A3	5 years trend
Other Process Uses of Carbonates	2A4	5 years trend
Chemical Industry	2B	5 years trend
Non-ferrous metals production (Aluminum, Magnesium, Lead, Zinc, Gold, Copper)	2C3, 2C4, 2C5, 2C6, 2C7	5 years trend
Non-Energy Products from Fuels and Solvent Use	2D	5 years trend
Electronic industry	2E	5 years trend
Product uses as substitutes of Ozone depleting substances	2F	5 years trend
Other Product Manufacture and Use	2G	5 years trend
Pulp and Paper industry	2H1	Trend based on combustion in pulp and paper industry
Food and Beverage industry	2H2	Trend based on combustion in food industry
Solid Waste Disposal	4A	5 years trend
Biological Treatment of Solid Waste	4B	5 years trend
Incineration and Open Burning of Waste	4C	5 years trend
Domestic Wastewater Treatment and Discharge	4D	No extrapolation
Industrial Wastewater Treatment and Discharge	4D	Trend based on combustion in chemical and pulp and paper industry

For the other sectors with relatively low contributions to global GHG emissions (around 8%) and for which it is still challenging to find reliable statistical data to update the activities up to t-1, their time series have been extended using relative changes in activity data compared to the last available year 2020 or 5-year trends.

## Data Records

The EDGAR Fast-Track emissions for the period 1970–2022 are available for all greenhouse gases and air pollutants (CO_2_, CH_4_, N_2_O, F-gases, SO_2_, NOx, CO, NMVOC, NH_3_, PM_10_, PM_2.5_, BC, OC) as time series in the format of Microsoft Excel files and gridmaps with 0.1 × 0.1 degree resolution in the format of .txt and .NetCDF files. The .xls files containing time series of emissions are individually produced for each gas (CO_2_, CH_4_, N_2_O, F-gases) and as aggregated GHGs. Each file contains emissions aggregated by IPCC 1996 and 2006 categories, for all countries (rows) and years (columns). A readme file and citation and references tab are also included. Emission gridmaps in.txt format include as columns the following variables: latitude, longitude, emission value expressed in ton substance/0.1degree × 0.1degree/year. Emission gridmaps in the format of .NetCDF files contain one variable with emission fluxes expressed in kg substance/m2/s. A single file is produced for each year for the gridmaps in .txt and .NetCDF files. All the data can be freely accessed via the EDGAR website at https://edgar.jrc.ec.europa.eu/report_2023, https://edgar.jrc.ec.europa.eu/dataset_ghg80 and https://edgar.jrc.ec.europa.eu/dataset_ap81 (last update: June 2024). We have applied the Fast-Track methodology described in this work to the latest EDGAR emission data from version 8. Data are registered at https://data.jrc.ec.europa.eu/dataset/b54d8149-2864-4fb9-96b9-5fd3a020c224^39^). EDGAR Fast Track emissions are downloadable online for each country (223 countries including international shipping and aviation) and source category (e.g. energy, industry, residential, transport, agriculture, etc.), for a total number of 40 aggregated processes following the IPCC 1996 and 2006 sector classification. The EDGAR Fast-Track emissions will be further improved for specific sectors or countries when new data will become available. New versions of EDGAR Fast-Track emissions will be periodically uploaded to the JRC EDGAR Repository.

## Technical Validation: Evaluation of the accuracy of the FT approach and discussion

In order to assess the accuracy of the FT approach, we calculated FT estimates for the 29 consecutive years spanning 1992-2020. These estimates project the official data forwards one and two years in each case (respectively denoted FT1 and FT2), and can be compared with the most detailed international statistics (e.g. IEA and FAO), hereafter named as official statistics. This means that for year *t*, we have the official value, the FT1 estimate (estimated from data up to *t*-1), and the FT2 estimate (estimated from data up to *t*-2). The time series can be of any of the EDGAR data outputs, including activity data or emissions data at the country level, country groups (e.g. EU27, regional/income grouping) or world totals and by substance and by activity sector. Correspondingly, the accuracy of the FT approach is dependent on the level of aggregation, activity sector and substance.

Our general approach calculates the FT error as the difference between official statistics and FT estimates. Implicitly this treats the official statistics as “true” measurements, whereas in fact the official emissions statistics have themselves a significant uncertainty – this is discussed in the following section.

### Accuracy of global GHG emissions

**Fig. 1:**
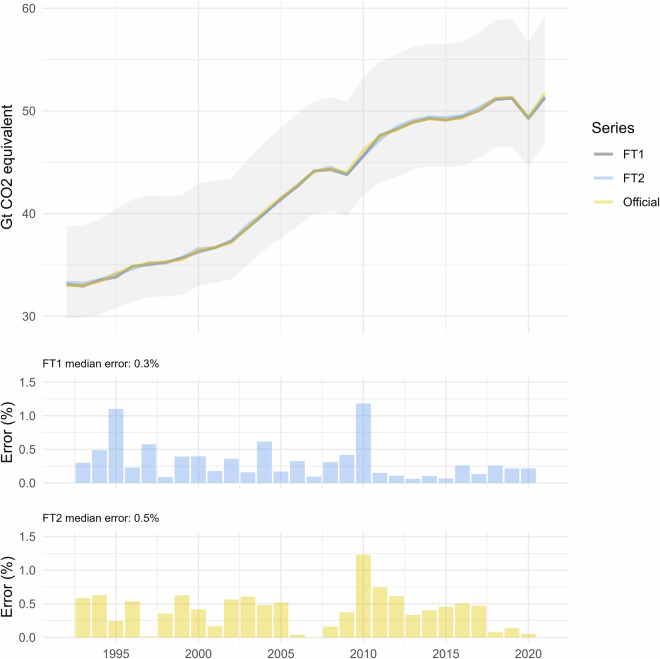
Global GHG emissions - comparison of official statistics with FT estimates. Shaded area in time series represents 90% confidence interval of official statistics due to uncertainties in activity data and emission factors.

At the highest level of aggregation, the FT estimates for global GHG emissions over all sectors are shown in Fig. 1, along with the official statistics and the resulting errors. The absolute pointwise errors here (calculated as the percentage difference between official statistics and FT estimates) are low, with a time-series median of 0.3% for FT1 and 0.5% for FT2. Typically, in the FT approach, FT2 error is slightly higher than FT1 error since it is projecting one year further ahead. No systematic patterns are found between the FT estimates and the official data (e.g. being always higher or lower than the reference). Accordingly with Solazzo *et al*.^33^ and Crippa *et al*.^35^, EDGAR global total GHG emissions are estimated with around ± 10% error, while the range of error for country level total CO_2_ emissions is between ± 4% and ± 35% (95% confidence interval). The FT estimates are well within the uncertainty bands. The shaded area in the time series plot in Fig. 1 shows the uncertainty in official statistics as a result of uncertainties in activity data and emission factors, and is estimated following the error propagation method described in^33^.

For few years (e.g. 1995, 1997, 2004, etc.), the FT methodology returns lowers errors for FT2 than FT1. FT2 is linked to the data from two years before, and will be more similar to trends and values from that time period. In some cases, there is an inflection of the time series, e.g. in 1997, which bucks the overall trend. The FT1 projection is more sensitive to these short-term fluctuations and presumes the trend may follow that inflection, but e.g. in the case of 1997 the following year returns to the more general trend. These simply represent uncertainties in the future trends, which will follow a random distribution. Although FT1 is on average more accurate, due to this randomness it will in some cases be less accurate than the FT2 projection.

### Accuracy of emission estimates by sector

#### Accuracy of combustion emissions

**Fig. 2:**
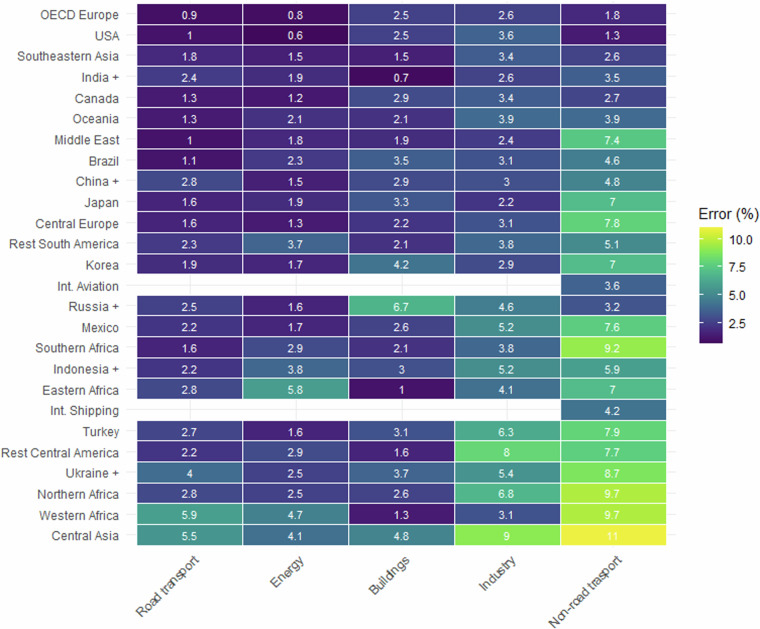
Median time series errors comparing official statistics against FT1 estimates, by combustion sectors and geographical region. Both axes are ordered by median error.

The accuracy of the FT approach varies with respect to the activity sector: this is due to the fact that (a) the method of estimation can be different for different sectors, and (b) the stability of the time series also varies between sectors. To show this in more depth, Fig. 2 shows the median errors (the median pointwise error over time) between official and FT1 estimates, for activity data time series, for combustion sectors and geographical regions (as defined in Table S1). Whereas road transport has the most accurate FT1 estimates on (on average, of the combustion sectors), non-road transport has the least accurate. Europe and USA have the most accurate projections on average. The highest error observed at this level of aggregation is for Central Asia in the non-road transport sector (11%).

**Fig. 3:**
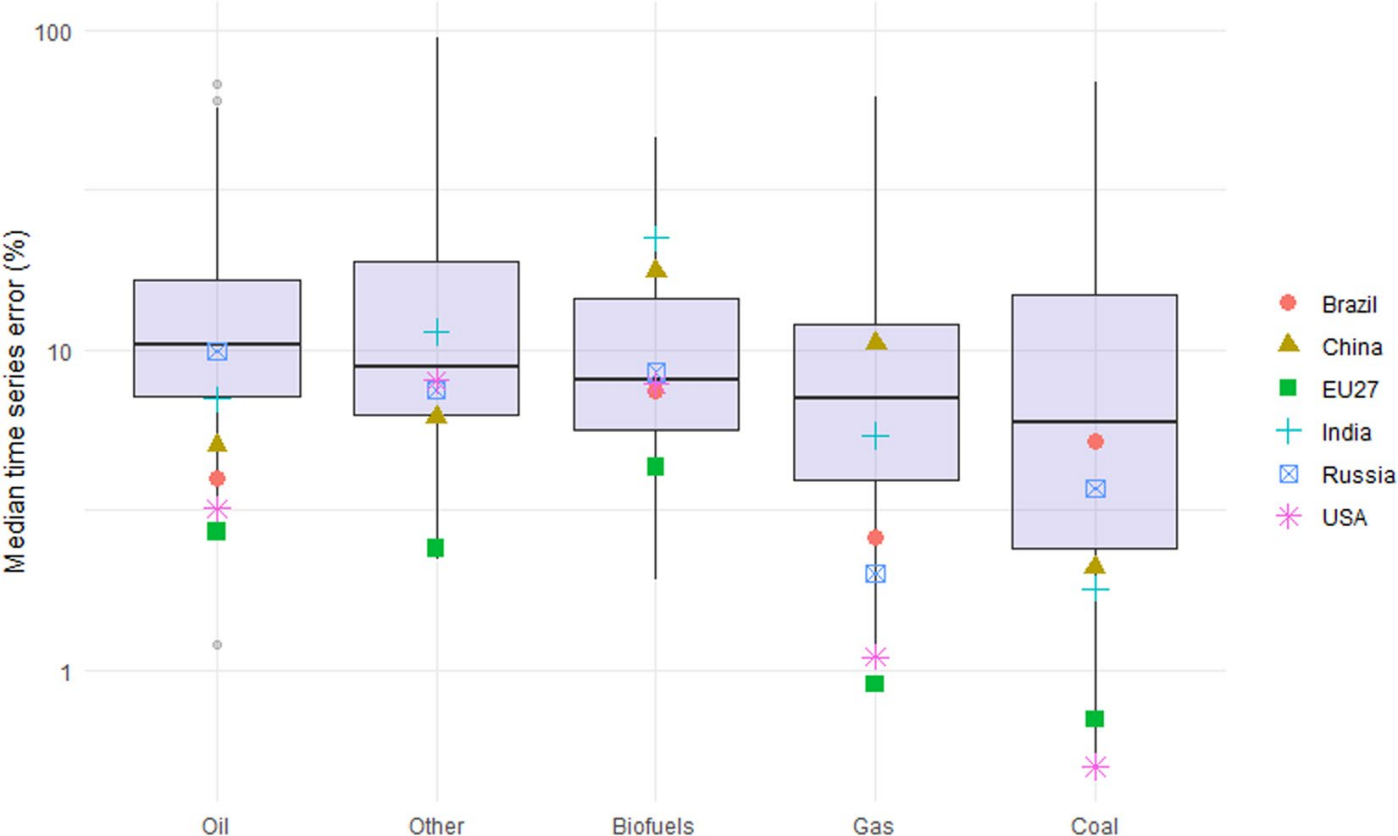
Median time series errors between official statistics and FT1, on activity data, by fuel type in the energy sector. Top-emitting countries plus EU27 are highlighted. Upper and lower box bounds represent 25th and 75th percentiles respectively. Note the log scale on the y-axis.

Figure 3 shows the median time series errors by fuel in the energy sector. The error distribution (over countries) varies between sectors: oil has the highest median error, whereas coal is typically more predictable. However, there is also a lot of variation between countries: the top-emitting countries typically have low errors, but there are exceptions: India and China tend to have higher errors, especially in the biofuels and gas sectors. The EU27 and USA tend to have the lowest errors of the top-emitting countries, with the EU27 ranging from 4.3% median error in biofuels down to 0.7% in gas, and the USA having a higher 8% error in the “Other” sector, but as low as 0.5% in coal.

#### Accuracy of agriculture emissions

**Fig. 4:**
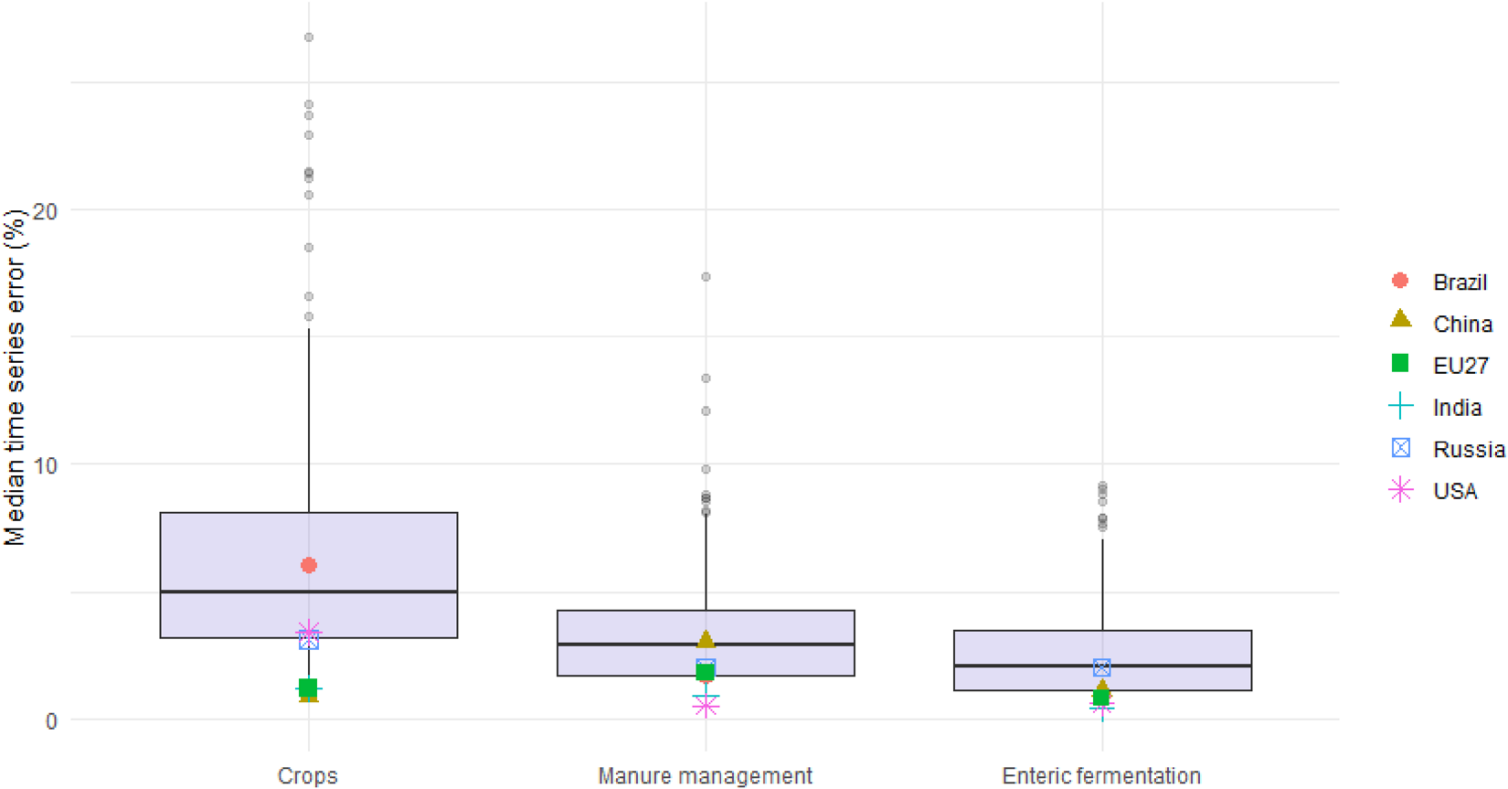
Median time series errors on activity data between official statistics and FT1, for agricultural sectors. Topemitting countries plus EU27 are highlighted, as well as some selected outliers. Upper and lower box bounds represent 25th and 75th percentiles respectively.

The same kind of sectoral evaluation is applied here to agricultural sectors as reported in Fig. 4. In this case it is evident that the FT approach is able to better predict the activity data in the livestock sectors (manure management and enteric fermentation) than in the crops sector. However, in all three sectors the median error is still relatively low for most countries. Top-emitting countries typically have low errors (e.g. EU27 has errors of 1.8% and 1.2% for manure management and crops, respectively), with the exception of Brazil in the crop sector (error 6%). Australia has a higher median error of 26.8% in the crop sector, which seems to be due to its fluctuating time series. Enteric fermentation has the lowest errors for top emitters, from 2% in Russia to 0.4% for India.

#### Accuracy of emissions by geographical region

**Fig. 5:**
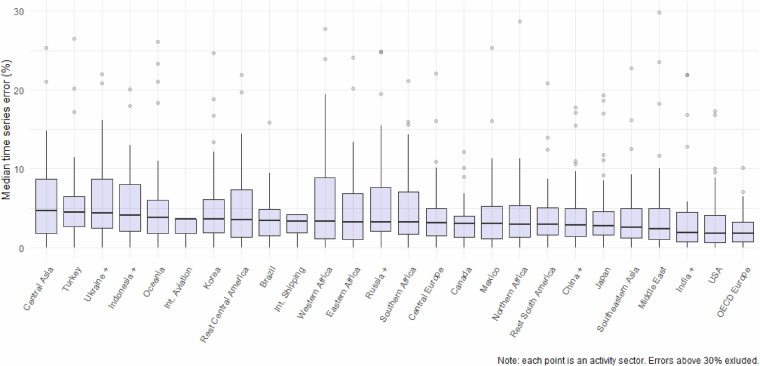
Median time series errors between official activity data statistics and FT1 estimates, by geographical region. Upper and lower box bounds represent 25th and 75th percentiles respectively. Outliers with median time series error above 30% (i.e. less than 1% of the sample) are not represented to improve the visibility of the figures. Note that each point represent an activity sector. Errors above 30% are excluded.

In this section we see how the accuracy of the FT approach varies between geographical regions. Intuitively, the reliability of the data is likely to be different between different countries, which each have different capacities for producing accurate national statistics. Figure 5 shows the error distributions for FT1 estimates, grouped by geographical region. The plot shows that on average, the OECD Europe region has the most reliable FT estimates (median 1.8% over all sectors), whereas the Central Asia region has the least reliable (median 4.7% over sectors). However, in all regions there is considerable variation between sectors, and this variation seems to be greater than between geographical regions.

Finally, Table 5 shows the FT1 and FT2 median errors for geographical groups and aggregated emissions sectors: here sectors are grouped into combustion and agriculture. The patterns are similar here: Europe and USA have some of the lowest errors. Combustion tends to have a slightly higher error than agriculture, although both are low (typically 1-2% for combustion and 2-3% for agriculture). A comprehensive view on country and sector specific errors associated with the Fast-Track approach is reported in Table S1.

**Table 5:** FT1 and FT2 errors by geographical region and aggregated activity sectors.

Geographical region	FT one-year		FT two-year	
	Combustion	Agriculture	Combustion	Agriculture
Brazil	0.9	1.1	1.6	1.7
Canada	1	1.1	0.8	2.5
Central Asia	2.5	1.8	4.4	2.2
Central Europe	0.8	1.5	0.8	3.1
China +	1.1	1.5	1.8	1.9
Eastern Africa	1.9	1.2	3.1	1.9
India +	1.1	0.3	2.2	0.7
Indonesia +	2.7	2.1	2.9	2.2
Int. Aviation	3.6		7	
Int. Shipping	4		8.7	
Japan	1.2	0.6	1.7	0.6
Korea	1.7	1.2	2.6	2.3
Mexico	1.3	1.8	2	3.3
Middle East	1.1	1.2	1.6	2.3
Northern Africa	1.4	1.1	2.2	1.6
Oceania	1.5	1.6	1.6	1.5
OECD Europe	0.7	0.8	1	1.7
Rest Central America	2.1	0.8	2.2	1.2
Rest South America	1.5	0.8	2.6	1.5
Russia +	1.4	1.3	2.4	2.1
Southeastern Asia	1.2	1	2.2	1.4
Southern Africa	1.8	1.5	3.6	3.1
Turkey	2.1	2.1	2.4	3.6
Ukraine +	1.1	2.2	1.9	3.2
USA	0.5	0.7	0.6	0.8
Western Africa	2.3	0.5	3.3	0.9

#### Absolute errors and trends

As well as focusing on pointwise percentage errors, we can measure the success of the FT approach in other ways. One angle of interest is to look at absolute errors in emissions estimates – this helps to understand which projections are contributing the most to errors at higher aggregations (such as global, regional, or cross-sectoral totals).

**Fig. 6:**
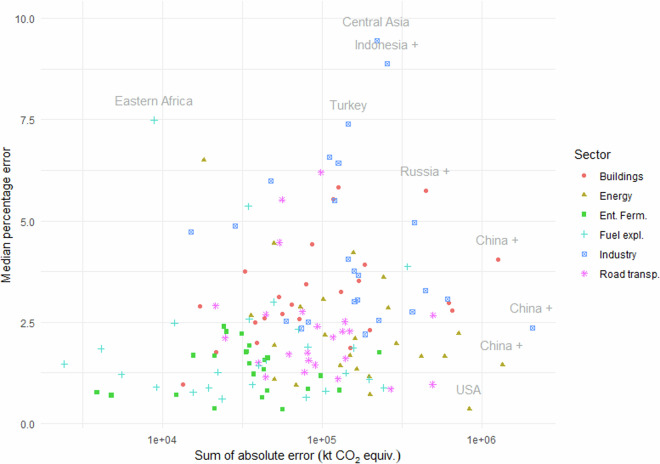
Median percentage time series error of total GHG emissions against sum of absolute error. Abbreviated sectors: “Ent. Ferm” = Enteric fermentation; “Fuel expl.” = Fuel exploitation; “Road transp.” = Road transport.

Figure 6 shows the median percentage time series error of total GHG emissions, against the sum of absolute error. In this plot, each point represents a time series the emissions of a country group in a particular sector. The six sectors in the plot represent those with the highest global GHG emissions. The plot reveals a slightly different story from those based purely on percentage error: for example, although the Central Asia industrial emissions have the highest median percentage error (9.4%), the greatest absolute error is in the China + time series, simply because its industrial emissions are much higher. This points to the fact that to improve global emissions FT estimates, it would be most productive to focus on improving the accuracy of China’s FT estimates in particular.

As a final validation, in this section we check the success of the FT approach to correctly predict the trend direction. At the global level, for GHG emissions over all sectors, the trend direction is correctly predicted in every tested year over 1994-2021, including those running against the overall trend (the financial crash in 2009 and the pandemic in 2020 – see again Fig. 1).

**Fig. 7:**
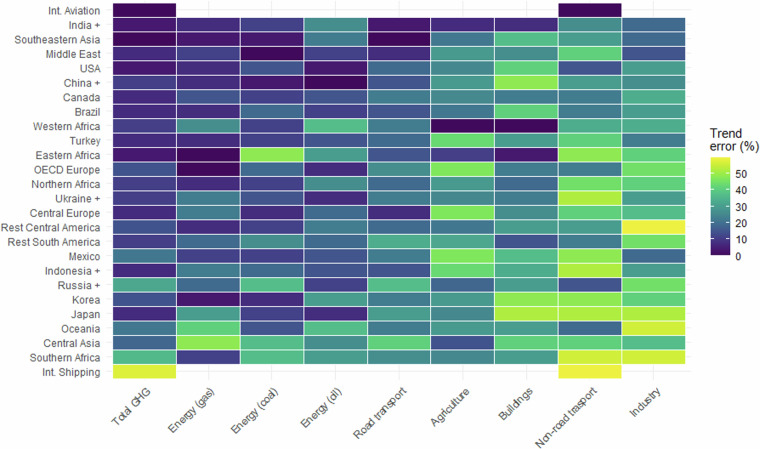
Errors in trend directions for FT1 estimates compared to official statistics, for aggregated sectors and geographical regions. See main text for further explanation.

Figure 7 shows the errors in the successful prediction of the trend direction at FT1. For each time series, representing the emissions of a given sector and geographical region, we checked the proportion that the FT1 trend direction agreed with the official statistics trend direction. For example, for each year in the time series, if the official statistics showed a positive trend and the FT1 estimate showed a positive trend, this was counted as a success, otherwise if the trend ran in the opposite direction it was counted as an error. The percentages in Fig. 7 therefore show the proportion of years in each time series where the FT1 and official trend directions were in opposite directions. Clearly, this metric is different but complementary to the pointwise errors investigated previously.

Figure 7 shows that whereas international aviation always has its trend direction correctly predicted (over the years investigated), international shipping is only correct around 50% of the time. In terms of trend direction, the errors for Europe are somewhat higher than for pointwise error.

## Usage Notes

This paper has presented a Fast-Track methodology for extending GHG emissions inventories up to the most recent years (*t*-1), based on a system of disaggregation and proxy variables. This is applied to the European Commission’s EDGAR database and enables it to provide detailed and timely emissions estimates that support monitoring the collective efforts to achieve global climate commitments under the Paris Agreement, but also national/international policy making and research. However, the methodology is equally applicable to any emissions inventory developed following an IPCC-based methodology.

The Fast-Track approach involves projecting emissions estimates forwards one or two years ahead of official statistics, and consequently comes with a margin of error. This error is of the order 0.3% and 0.5% for one and two-year projections respectively, when projecting global GHG emissions. However, at the more disaggregated levels the error is dependent on the activity sector, as well as the country. Specifically, Europe and USA tend to have quite low errors, presumably due to the reliability or level of detail of statistics. On the other hand, Central Asia has some of the higher errors on average. Top emitters tend to have a lower percentage error but due to their size can contribute a significant error proportion to total GHG projections. Therefore, in order to improve global emissions FT estimates, it would be most productive to focus on improving the accuracy of China’s FT estimates and of other top emitting economies. We note that Fast-Track error margins are typically well within the emissions confidence intervals which are calculated using uncertainties in activity data and emission factors. Therefore, EDGAR data users need to be aware of the higher uncertainty of the emissions for the years t-2 and t-1 compared to the historic data which are entirely based on consolidated statistics.

In order to allow a straightforward use of the EDGAR Fast-Track emissions by inventory developers and atmospheric modellers, each emitting sector has been mapped with a sector description and the standard Intergovernmental Panel on Climate Change^11,12^ classification and definition of source categories. Similarly, countries are identified with their name, regional belonging and International Organization for Standardization (ISO 3166-1 alpha-3 standard) codes in order to allow a clear and unique identification by any user. Country names are consistent with the Interinstitutional Style Guide of the European Commission available at http://publications.europa.eu/code/en/en-370100.htm, the “Short name” definition listed in the “List of countries, territories and currencies” table at http://publications.europa.eu/code/en/en-5000500.htm has been used (updated on 04/07/2023).

## Supplementary information

Supplementary Information

## Data Availability

Most of the Fast-Track emission computation methodology and data processing has been done using the software Python version 3.6. Further computations, such as mapping sectors and countries have been performed using Microsoft Access 2010. The implementation of the EDGAR Fast-Track methodology into the Emissions Database for Global Atmospheric Research has been developed using a dedicated EDGAR development tool of the Joint Research Centre named EOLO based on Php and Microsoft SQL Server. This system cannot be accessed outside the institution but further details can be provided upon request. Uncertainty emission calculation and data validation have been performed with R.
