# Supplementary material for: Global up-to date emissions using the EDGAR Fast-Track methodology
Source: Sci Data. 2025 Jul 16;12:1243. doi: 10.1038/s41597-025-04806-2 (PMC12267624; doi:10.1038/s41597-025-04806-2)
Supplement: Supplementary file 1 — Supplementary Information [file 41597_2025_4806_MOESM1_ESM.docx]

Supplementary Information for manuscript:

**Global up-to date emissions using the EDGAR Fast-Track methodology**

Diego Guizzardi^1^, William Becker^2^, Federico Pagani^1^, Marilena Muntean^1^, Monica Crippa^1^

^1^European Commission, Joint Research Centre (JRC), Ispra, Italy

^2^Unisystems S.A., Milan, Italy


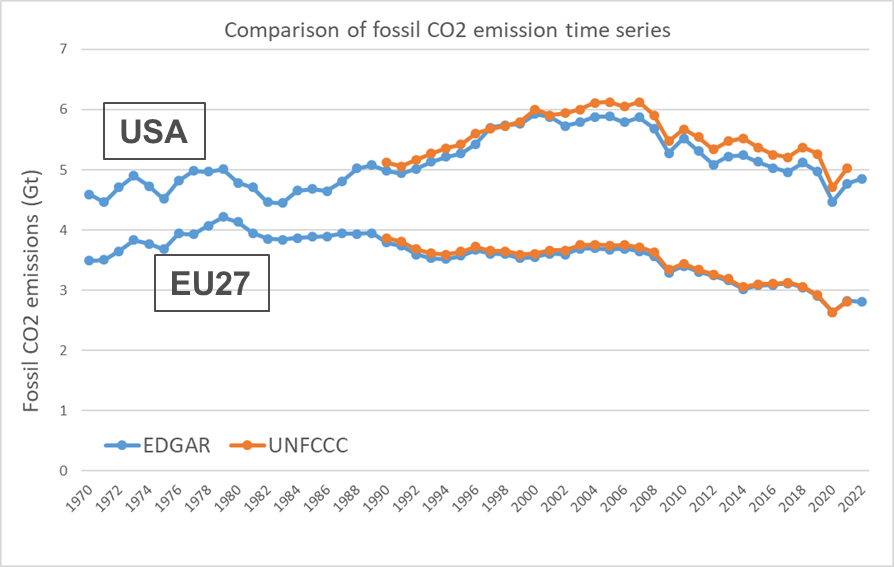


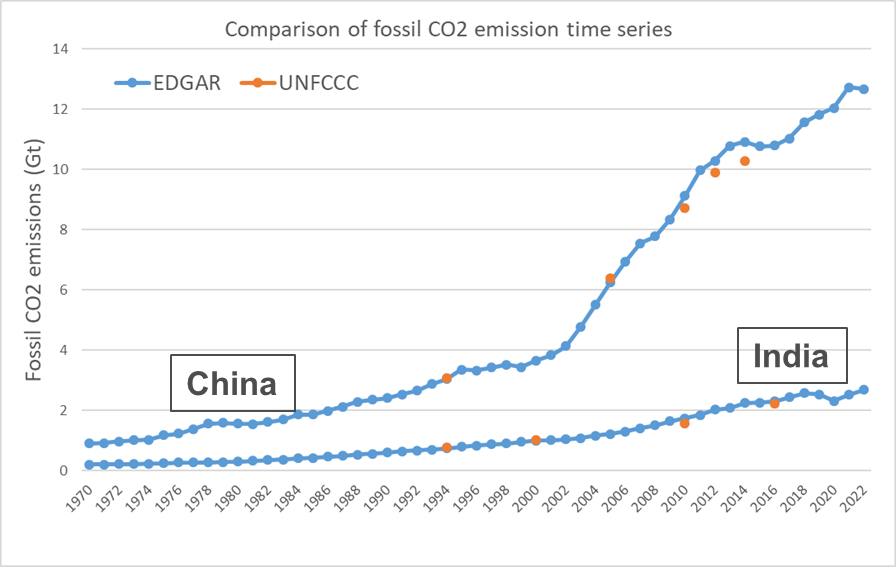


Figure S1 – Comparison between EDGAR and National Emission Inventories (NEIs) emission reporting (labelled as UNFCCC) for fossil CO_2_. The top panel shows the comparison for two Annex-I countries, while the bottom panel it is for two selected non-Annex I countries, showing the very limited data availability for the latter for the UNFCCC reporting.

Table S1: Median percentage errors by country and major sectors of official GHG emissions statistics compared to FT1 estimates. The “Uncertainty” column is indicative and refers to the median uncertainty of official emissions statistics due to uncertainties in activity data and emission factors.

| **Country** | **Region** | **Energy** | **Industry** | **Buildings** | **Road transport** | **Non-road transport** | **Uncertainty** |
| --- | --- | --- | --- | --- | --- | --- | --- |
| Aruba | Rest Central America | 5.2 | 10 | 11.9 | 7.8 | 9.7 | 9 |
| Afghanistan | India + | 13.3 | 25.4 | 11.3 | 17.2 | 19.1 | 43 |
| Angola | Southern Africa | 9.3 | 18.5 | 4.5 | 8.7 | 12.2 | 106 |
| Anguilla | Rest Central America | 4.1 | 4.4 | 8.6 | 7 |  | 15 |
| International aviation | Int. Aviation | |  |  |  | 3.6 | 6 |
| Albania | Central Europe | 21.9 | 23.4 | 12.1 | 4.9 | 7 | 16 |
| Antigua and Barbuda | Rest Central America | 4.9 | 2.5 | 2.7 | 2.5 |  | 7 |
| United Arab Emirates | Middle East | 2.6 | 4.3 | 5.7 | 3.5 | 6.3 | 30 |
| Argentina | Rest South America | 2.4 | 6.5 | 2.9 | 3.5 | 4 | 29 |
| Armenia | Russia + | 11 | 16.1 | 7.6 | 10.8 |  | 10 |
| Antigua & Barbuda | Rest Central America | 6.3 | 10.2 | 10.4 | 7.2 | 8.2 | 21 |
| Australia | Oceania | 1.6 | 3.4 | 1.9 | 1.4 | 3.5 | 13 |
| Austria | OECD Europe | 4 | 3 | 3.8 | 2 | 4.2 | 7 |
| Azerbaijan | Russia + | 5.3 | 20.4 | 9.1 | 12 | 10.3 | 8 |
| Burundi | Eastern Africa | 10.7 | 13.4 | 1.4 | 8.1 | 11 | 54 |
| Belgium | OECD Europe | 3.4 | 4.2 | 5.8 | 3.3 | 5.8 | 7 |
| Benin | Western Africa | 15.3 | 13.3 | 4.7 | 7.9 |  | 34 |
| Burkina Faso | Western Africa | 7 | 10.8 | 1.6 | 7 | 10.9 | 57 |
| Bangladesh | India + | 4.3 | 14.5 | 2.1 | 5.1 | 5.2 | 27 |
| Bulgaria | Central Europe | 1.8 | 4 | 4.5 | 4.3 | 15 | 10 |
| Bahrain | Middle East | 3.9 | 11 | 5.4 | 3.3 | 16.5 | 78 |
| Bahamas | Rest Central America | 9 | 8.7 | 11.5 | 10.1 | 8.9 | 21 |
| Bosnia & Herzegovina | Central Europe | 4.7 | 11.8 | 12 | 7.4 |  | 13 |
| Belarus | Ukraine + | 2.8 | 6.8 | 4 | 7.6 | 10.8 | 12 |
| Belize | Rest Central America | 8.2 | 8.8 | 6.7 | 6.7 | 11.5 | 43 |
| Bermuda | Rest Central America | 8.8 | 13.6 | 11.6 | 8.3 | 12.5 | 12 |
| Bolivia | Rest South America | 9.6 | 7.7 | 3 | 4.3 | 5.3 | 38 |
| Brazil | Brazil | 2.7 | 2.5 | 2.5 | 1.6 | 4.4 | 32 |
| Barbados | Rest Central America | 6 | 10.7 | 10.8 | 7.5 | 7.5 | 19 |
| Brunei | South Eastern Asia | 12.3 | 11.7 | 13 | 4.1 |  | 56 |
| Bhutan | India + | 8 | 14.2 | 1.1 | 6.2 | 11.2 | 35 |
| Botswana | Southern Africa | 6.4 | 9.1 | 6.2 | 4.5 | 10.1 | 38 |
| Central African Republic | Western Africa | 8.9 | 10 | 1.3 | 7.6 | 9.9 | 57 |
| Canada | Canada | 1.1 | 2.3 | 2.6 | 1.3 | 2.3 | 9 |
| Switzerland | OECD Europe | 3.4 | 4 | 2.9 | 3.2 | 9.8 | 6 |
| Chile | Rest South America | 6.3 | 8.2 | 3.2 | 2.5 | 9 | 19 |
| China | China + | 1.4 | 2.4 | 4 | 3.5 | 4.3 | 14 |
| Cote d'Ivoire | Western Africa | 16.4 | 13.3 | 6.5 | 7.6 | 10.8 | 35 |
| Cameroon | Western Africa | 13.6 | 7.5 | 2.5 | 5 | 8 | 67 |
| Congo - Kinshasa | Western Africa | 9.5 | 3.6 | 1.7 | 8.4 |  | 51 |
| Congo - Brazzaville | Western Africa | 12.6 | 33.3 | 4.7 | 9.6 | 18.6 | 151 |
| Cook Islands | Oceania | 10.8 | 9 | 17.5 | 10 | 15.7 | 12 |
| Colombia | Rest South America | 16.8 | 8.6 | 6.3 | 3.1 | 5.7 | 31 |
| Comoros | Eastern Africa | 8.2 | 9 | 3.1 | 7.5 | 10.7 | 33 |
| Cape Verde | Western Africa | 8 | 9.5 | 5.6 | 5.9 | 9 | 15 |
| Costa Rica | Rest Central America | 46.4 | 5.7 | 6.2 | 2.7 | 8.3 | 27 |
| Cuba | Rest Central America | 3.7 | 12.6 | 9.4 | 7.4 | 11.3 | 27 |
| Cayman Islands | Rest Central America | 6.8 | 10.3 | 8.2 | 6.6 | 7.8 | 10 |
| Cyprus | Central Europe | 3.6 | 8.7 | 8.6 | 3.3 |  | 5 |
| Czechia | Central Europe | 1.8 | 4.5 | 3.6 | 4.1 | 9.9 | 9 |
| Germany | OECD Europe | 0.6 | 2 | 4.3 | 1.1 | 4.4 | 6 |
| Djibouti | Eastern Africa | 8.8 | 10 | 6.2 | 6.3 | 10.8 | 33 |
| Dominica | Rest Central America | 8.4 | 10.3 | 12.2 | 8.3 | 11.7 | 28 |
| Denmark | OECD Europe | 2 | 5.6 | 2.1 | 3.2 | 7 | 9 |
| Dominican Republic | Rest Central America | 6.4 | 8 | 5.3 | 7.4 | 7.3 | 18 |
| Algeria | Northern Africa | 3 | 4.2 | 3.6 | 1.3 | 14.8 | 55 |
| Ecuador | Rest South America | 10.3 | 6.1 | 6.5 | 2.7 | 8.1 | 35 |
| Egypt | Northern Africa | 2.8 | 9.1 | 2.9 | 3.3 | 7.8 | 23 |
| Eritrea | Eastern Africa | 7.2 | 9.6 | 3.1 | 12.1 | 34.6 | 54 |
| Western Sahara | Northern Africa | 5.9 | 5.7 | 7.9 | 4.4 | 5.7 | 34 |
| Spain | OECD Europe | 1 | 6.2 | 3.8 | 1.6 | 4.1 | 7 |
| Estonia | Central Europe | 4.7 | 9.5 | 5.2 | 4.1 | 10.1 | 15 |
| Ethiopia | Eastern Africa | 15 | 7.9 | 1.4 | 5 | 11 | 58 |
| Finland | OECD Europe | 2.9 | 5.7 | 3.4 | 1.8 | 2.9 | 9 |
| Fiji | Oceania | 14.4 | 10.9 | 15.8 | 7.8 | 10.7 | 27 |
| Falkland Islands | Rest South America | 8.3 | 15.4 | 15 | 6.1 | 10 | 12 |
| France | OECD Europe | 3.6 | 3.2 | 3.1 | 1.2 | 2.3 | 9 |
| Faroe Islands | OECD Europe |  |  | 0 |  |  | 27 |
| Gabon | Western Africa | 10.8 | 8.5 | 2.2 | 8.4 | 9.8 | 160 |
| United Kingdom | OECD Europe | 1.6 | 3.1 | 2.9 | 1.1 | 3.1 | 7 |
| Georgia | Russia + | 14.9 | 14.3 | 9.2 | 9.5 | 25.2 | 10 |
| Ghana | Western Africa | 19.2 | 5 | 6.9 | 6.4 | 9.5 | 32 |
| Gibraltar | OECD Europe | 7.2 |  | 0 | 6 |  | 4 |
| Guinea | Western Africa | 7.5 | 5.9 | 1.8 | 4.2 | 7.9 | 40 |
| Guadeloupe | Rest Central America | 4.3 | 8.9 | 7.8 | 5.2 | 6.8 | 15 |
| Gambia | Western Africa | 7.5 | 7.5 | 2.6 | 5.9 | 8.1 | 40 |
| Guinea-Bissau | Western Africa | 7 | 6.5 | 1.1 | 4.3 | 9.2 | 45 |
| Equatorial Guinea | Western Africa | 8.2 | 20.2 | 6.2 | 7.5 | 6.6 | 122 |
| Greece | OECD Europe | 2.5 | 6.2 | 4.9 | 2 | 6.4 | 8 |
| Grenada | Rest Central America | 10.3 | 10.6 | 10.3 | 10.2 | 7.9 | 25 |
| Greenland | OECD Europe | 4.6 | 9.2 | 3.2 | 4.6 | 6.2 | 7 |
| Guatemala | Rest Central America | 9.3 | 6.4 | 1.9 | 4 | 6.8 | 30 |
| French Guiana | Rest South America | 6 | 5.3 | 7.7 | 6.2 | 5.3 | 20 |
| Guam | Oceania |  |  | 0.4 |  |  | 64 |
| Guyana | Rest South America | 5.8 | 12.6 | 8.1 | 4.2 | 7.1 | 73 |
| Hong Kong SAR China | China + | 1.2 | 9.5 | 5.4 | 9 | 13.7 | 12 |
| Honduras | Rest Central America | 13.5 | 10.1 | 6.7 | 3.4 | 7.7 | 31 |
| Croatia | Central Europe | 8 | 3.5 | 3.1 | 4.6 | 4.6 | 8 |
| Haiti | Rest Central America | 10.6 | 12.6 | 2 | 5.6 | 8.4 | 51 |
| Hungary | Central Europe | 3.8 | 2.9 | 3.4 | 5 | 7.3 | 9 |
| Indonesia | Indonesia + | 4.4 | 9.2 | 3.2 | 2.3 | 6.1 | 28 |
| India | India + | 1.9 | 3.5 | 3 | 1.9 | 4.6 | 20 |
| Ireland | OECD Europe | 2.6 | 4.2 | 2.8 | 2.8 | 7.5 | 18 |
| Iran | Middle East | 1.9 | 4.4 | 2.7 | 2.8 | 18.8 | 36 |
| Iraq | Middle East | 9.5 | 10.5 | 10.3 | 8 | 14.8 | 81 |
| Iceland | OECD Europe | 36.3 | 5.9 | 6.5 | 3 | 16.4 | 10 |
| Israel | Middle East | 2.4 | 17.1 | 7.1 | 4 | 5.6 | 10 |
| Italy | OECD Europe | 0.9 | 2.4 | 4 | 2.1 | 2.4 | 5 |
| Jamaica | Rest Central America | 5.8 | 15.5 | 8.4 | 6 | 5.4 | 9 |
| Jordan | Middle East | 4.7 | 4 | 7.2 | 4.3 | 11.5 | 13 |
| Japan | Japan | 1.7 | 2.2 | 3.5 | 1.6 | 6.7 | 6 |
| Kazakhstan | Central Asia | 6.4 | 11.8 | 9.4 | 8.8 | 18.9 | 12 |
| Kenya | Eastern Africa | 18.7 | 10 | 3.2 | 6.9 | 13.6 | 49 |
| Kyrgyzstan | Central Asia | 12.6 | 28.2 | 20.8 | 16.8 |  | 17 |
| Cambodia | South Eastern Asia | 13.8 | 8.1 | 4.5 | 6.7 | 7.3 | 23 |
| Kiribati | Oceania | 7.7 | 11.2 | 10.7 | 8 | 12.5 | 17 |
| St. Kitts & Nevis | Rest Central America | 8.3 | 10.5 | 11 | 9 | 11.9 | 29 |
| South Korea | Korea | 1.6 | 5.2 | 6.4 | 1.9 | 6.7 | 6 |
| Kuwait | Middle East | 5.2 | 10 | 9 | 4.6 |  | 53 |
| Laos | South Eastern Asia | 11 | 21.8 | 2.2 | 6.8 | 3.1 | 27 |
| Lebanon | Middle East | 8.3 | 25.5 | 18.8 | 8.8 |  | 13 |
| Liberia | Western Africa | 8.2 | 10.8 | 1.7 | 4.1 | 7.6 | 54 |
| Libya | Northern Africa | 3.8 | 6.8 | 5.5 | 5.5 | 9.6 | 79 |
| St. Lucia | Rest Central America | 4.4 | 10.8 | 9.7 | 6.3 | 6.9 | 20 |
| Sri Lanka | India + | 11.6 | 11.5 | 3 | 4.3 | 10.2 | 19 |
| Lesotho | Southern Africa | 6.1 | 9.3 | 2.3 | 7.8 | 10.4 | 50 |
| Lithuania | Central Europe | 7.4 | 7 | 7.2 | 6.9 | 6.5 | 11 |
| Luxembourg | OECD Europe | 11.5 | 7.1 | 4.1 | 4.2 | 14.6 | 5 |
| Latvia | Central Europe | 5.6 | 10.7 | 6.3 | 6 | 10 | 13 |
| Macao SAR China | China + | 10.9 | 8.9 | 14.2 | 8.9 | 10.4 | 9 |
| Morocco | Northern Africa | 3.8 | 5 | 3.4 | 3.4 | 8 | 25 |
| Moldova | Ukraine + | 6 | 9.8 | 11 | 10.9 | 27.7 | 9 |
| Madagascar | Eastern Africa | 12.5 | 14.1 | 2.5 | 3.9 | 15.7 | 48 |
| Maldives | India + | 14.9 | 15.5 | 16.5 | 11.4 | 12.3 | 11 |
| Mexico | Mexico | 0.9 | 5.5 | 3.8 | 2.1 | 6.7 | 8 |
| North Macedonia | Central Europe | 4.4 | 10.4 | 12.1 | 6.6 | 5.8 | 9 |
| Mali | Western Africa | 4.4 | 9.6 | 2.2 | 4 | 7.8 | 59 |
| Malta | Central Europe | 8.1 | 16 | 11.9 | 8 | 10.2 | 4 |
| Myanmar (Burma) | South Eastern Asia | 11.6 | 10.6 | 4.2 | 8.5 | 6.5 | 24 |
| Mongolia | China + | 7.4 | 12.6 | 20.8 | 5.6 | 17.1 | 40 |
| Mozambique | Southern Africa | 21.7 | 9.4 | 3.1 | 5.5 | 8.2 | 36 |
| Mauritania | Western Africa | 4.4 | 7 | 3 | 4.9 | 8.2 | 49 |
| Montserrat | Rest Central America | 7.4 | 13.8 | 20.2 | 7 | 12.6 | 41 |
| Martinique | Rest Central America | 4.7 | 6.8 | 3.7 | 4.8 | 6.3 | 12 |
| Mauritius | Eastern Africa | 10.9 | 5.6 | 2.5 | 4.1 | 8 | 16 |
| Malawi | Southern Africa | 18.1 | 13.2 | 2.2 | 7.3 | 13.5 | 32 |
| Malaysia | South Eastern Asia | 4 | 4.4 | 14.3 | 2.8 | 9 | 23 |
| Mayotte | Eastern Africa |  |  |  |  |  | 70 |
| Namibia | Southern Africa | 63 | 4.7 | 4.7 | 5.2 | 7 | 51 |
| New Caledonia | Oceania | 10.3 | 12.4 | 11.6 | 4.7 | 11.7 | 8 |
| Niger | Western Africa | 8.6 | 22.4 | 2.1 | 9 | 2.6 | 63 |
| Nigeria | Western Africa | 11.1 | 11.7 | 2.4 | 9.8 | 18.1 | 101 |
| Nicaragua | Rest Central America | 4.8 | 6.5 | 2.1 | 3.6 | 10.7 | 48 |
| Niue | Oceania |  |  | 0 |  |  | 47 |
| Netherlands | OECD Europe | 1.1 | 3.2 | 3 | 2.4 | 5.3 | 6 |
| Norway | OECD Europe | 11.9 | 3.2 | 5.2 | 2.6 | 8.4 | 11 |
| Nepal | India + | 42.4 | 19.6 | 2.6 | 5.6 |  | 31 |
| New Zealand | Oceania | 7.6 | 5.3 | 3.4 | 1.8 | 4.9 | 24 |
| Oman | Middle East | 4.8 | 11 | 14.2 | 5.9 | 20 | 87 |
| Pakistan | India + | 3.6 | 4.2 | 2.4 | 3.6 | 4.9 | 29 |
| Panama | Rest Central America | 18.7 | 9.7 | 7.7 | 2.6 | 18.7 | 21 |
| Peru | Rest South America | 7.8 | 6.2 | 4.6 | 1.8 | 24.8 | 24 |
| Philippines | South Eastern Asia | 2.7 | 7.2 | 3.3 | 2.7 | 7.6 | 15 |
| Palau | Oceania | 10 | 13.1 | 14 | 16.5 | 15.1 | 11 |
| Papua New Guinea | Indonesia + | 10.6 | 8.6 | 8.5 | 8.2 | 10.9 | 28 |
| Poland | Central Europe | 1.1 | 5.2 | 4.6 | 2.2 | 8.7 | 10 |
| Puerto Rico | Rest Central America | 6.4 | 20.5 | 9.9 | 4.4 | 9.9 | 10 |
| North Korea | Korea | 4.7 | 2.1 | 2.4 | 9.3 |  | 18 |
| Portugal | OECD Europe | 4.5 | 5.7 | 5 | 2 | 5.9 | 6 |
| Paraguay | Rest South America | 7.6 | 12.8 | 2.6 | 8.1 | 6.2 | 49 |
| French Polynesia | Oceania | 8.5 | 6.8 | 13.3 | 6.1 | 10.1 | 10 |
| Qatar | Middle East | 5.8 | 8.5 | 5.9 | 6.8 |  | 66 |
| Reunion | Eastern Africa | 5.8 | 6.5 | 4.5 | 4.7 | 9.3 | 9 |
| Romania | Central Europe | 3 | 4.9 | 4.6 | 4.3 | 11.4 | 9 |
| Russia | Russia + | 2.3 | 4.8 | 5.8 | 2.7 | 3.5 | 11 |
| Rwanda | Eastern Africa | 10.2 | 7.3 | 1.3 | 4.8 |  | 47 |
| Saudi Arabia | Middle East | 1.6 | 5.2 | 4.9 | 2.9 | 3.7 | 29 |
| Serbia and Montenegro | Central Europe | 3 | 10.1 | 11.8 | 6.4 | 11.2 | 10 |
| Sudan | Eastern Africa | 11.9 | 17.7 | 3 | 6.8 | 16.9 | 57 |
| International shipping | Int. Shipping |  |  |  |  | 4 | 20 |
| Senegal | Western Africa | 6.7 | 15.8 | 5.6 | 6.1 | 12.6 | 38 |
| Singapore | South Eastern Asia | 6.3 | 5.6 | 8.7 | 4.5 | 9.7 | 8 |
| St. Helena | Western Africa | 5.1 | 9.1 | 7.3 | 4.4 |  | 12 |
| Solomon Islands | Oceania | 7.2 | 6.2 | 9.7 | 6.5 | 9.1 | 19 |
| Sierra Leone | Western Africa | 10.2 | 7.1 | 1.5 | 7.3 | 10.6 | 39 |
| El Salvador | Rest Central America | 9 | 8 | 8.8 | 3.7 |  | 24 |
| Somalia | Eastern Africa | 6.5 | 7.1 | 1.1 | 3.7 | 9.1 | 55 |
| St. Pierre & Miquelon | USA | 11.3 | 13.8 | 14.8 | 9.5 | 12.5 | 6 |
| Sao Tome & Principe | Western Africa | 6.5 | 8.6 | 3.3 | 6.5 | 9.1 | 24 |
| Suriname | Rest South America | 15.9 | 12.1 | 12.4 | 6.7 | 13.5 | 17 |
| Slovakia | Central Europe | 3.3 | 4.8 | 3 | 5.1 | 15.8 | 9 |
| Slovenia | Central Europe | 4.2 | 5.2 | 9.9 | 2.9 | 8.9 | 13 |
| Sweden | OECD Europe | 6.2 | 4.3 | 3.4 | 3.6 | 3.9 | 10 |
| Eswatini | Southern Africa | 14.3 | 10.2 | 7.5 | 7.8 |  | 38 |
| Seychelles | Eastern Africa | 7.4 | 6.1 | 13.7 | 6.5 | 13.2 | 9 |
| Syria | Middle East | 3.5 | 6.3 | 8 | 5.9 | 23.1 | 32 |
| Turks & Caicos Islands | Rest Central America | 7.1 | 10.1 | 11.3 | 8.6 | 9.6 | 18 |
| Chad | Western Africa | 7.3 | 10.3 | 1.4 | 7.5 | 11.2 | 66 |
| Togo | Western Africa | 41 | 44.3 | 4.4 | 26.6 |  | 49 |
| Thailand | South Eastern Asia | 1.8 | 4.9 | 4.3 | 2.9 | 4.5 | 12 |
| Tajikistan | Central Asia | 18.7 |  | 11 | 18.2 |  | 14 |
| Turkmenistan | Central Asia | 17.1 | 22.8 | 9.4 | 2.4 | 24.3 | 15 |
| Timor-Leste | South Eastern Asia | 8.5 | 5.3 | 3.1 | 8.6 | 12.5 | 43 |
| Tonga | Oceania | 10.5 | 10.5 | 10.4 | 10.7 | 12.3 | 22 |
| Trinidad & Tobago | Rest Central America | 4.7 | 6.2 | 10.2 | 11.7 | 8.4 | 24 |
| Tunisia | Northern Africa | 6.8 | 3.8 | 2.8 | 3.3 | 54.3 | 24 |
| Turkey | Turkey | 1.9 | 7.4 | 4.4 | 2.7 | 7 | 8 |
| Taiwan | China + | 0.8 | 2.8 | 2.9 | 1.9 | 7.6 | 8 |
| Tanzania | Southern Africa | 16.1 | 5.9 | 2.1 | 7.5 | 9.3 | 51 |
| Uganda | Eastern Africa | 10.9 | 5.7 | 0.6 | 4.1 | 4.3 | 56 |
| Ukraine | Ukraine + | 3 | 4.3 | 4.6 | 4.4 | 10 | 15 |
| Uruguay | Rest South America | 61.9 | 7.7 | 4.9 | 3.5 | 20.9 | 49 |
| United States | USA | 0.4 | 3.1 | 2.8 | 1 | 1.4 | 6 |
| Uzbekistan | Central Asia | 4.8 | 10.4 | 5.9 | 5.3 | 8.4 | 12 |
| St. Vincent & Grenadines | Rest Central America | 8.4 | 11.1 | 12.5 | 10.5 | 11.2 | 28 |
| Venezuela | Rest South America | 6.4 | 9.3 | 9.7 | 7.7 | 21.8 | 29 |
| British Virgin Islands | Rest Central America | 6.5 | 12 | 12.7 | 7.8 | 11.6 | 10 |
| U.S. Virgin Islands | Rest Central America |  |  | 2.8 |  |  | 48 |
| Vietnam | South Eastern Asia | 2.9 | 6.4 | 5.4 | 2.9 | 8.2 | 21 |
| Vanuatu | Oceania | 10.6 | 8.5 | 10.7 | 9.2 | 13.9 | 45 |
| Samoa | Oceania | 7.5 | 7.1 | 11.7 | 7.9 | 10.1 | 19 |
| Yemen | Middle East | 7.5 | 10.8 | 6.7 | 7.5 |  | 43 |
| South Africa | Southern Africa | 2.8 | 8.2 | 9.3 | 2.2 | 8 | 17 |
| Zambia | Southern Africa | 11.2 | 9.5 | 1.6 | 5.1 | 8.5 | 78 |
| Zimbabwe | Southern Africa | 12.3 | 16.9 | 3.9 | 12 | 10.5 | 36 |
